# Deaths in Philadelphia from March 23d to April 20th, 1850

**Published:** 1850-05

**Authors:** James Aitken Meigs

**Affiliations:** Student of Medicine


*Deaths in Philadelphia from March 23d to April 20th,* 1850. Reported by Mr. JAMES AITKEN MEIGS, Student of Medicine.

| Diseases. | Ad'ts | Chil. | Diseases. | Ad'ts | Chil. |
|---|---|---|---|---|---|
| Abortion, | 1 | 0 | Hemorrhage, | 0 | 3 |
| Abscess, | 0 | 1 | " from stomach, | 1 | 0 |
| " of lungs | 2 | 0 | Hernia, | 1 | 1 |
| Anæmia, | 0 | 1 | " umbilical, | 1 | 0 |
| Apoplexy, | 4 | 0 | Hydrocele, | 0 | 1 |
| Burns, | 1 | 2 | Inflammation, general, | 1 | 0 |
| Cancer, | 1 | 0 | " of brain, | 0 | 11 |
| " of lungs, | 1 | 0 | " breast, | 0 | 2 |
| " of mouth, | 1 | 0 | " bronchi, | 3 | 14 |
| " stomach, | 1 | 0 | " larynx, | 0 | 3 |
| " uterus, | 1 | 0 | " liver, | 1 | 0 |
| Casualties, | 4 | 3 | " lungs, | 12 | 22 |
| Cholera infantum, | 0 | 1 | " œsophagus, | 1 | 0 |
| Compression of brain, | 0 | 1 | " peritoneum, | 2 | 0 |
| Congestion of lungs, | 2 | 5 | " pericardium, | 0 | 1 |
| " brain, | 5 | 2 | " pleura, | 2 | 0 |
| Convulsions, | 2 | 28 | " stom. & bowels, | 4 | 5 |
| " puerperal, | 1 | 0 | " tonsils, | 1 | 0 |
| Croup, | 0 | 15 | Intemperance & exposure, | 5 | 0 |
| Cyanosis, | 0 | 1 | Ileus, | 1 | 0 |
| Debility, | 3 | 7 | Jaundice, | 1 | 0 |
| Dentition, | 0 | 3 | Malformation of heart, | 0 | 1 |
| Diarrhœa, | 1 | 1 | Malignant disease of eye, | 0 | 1 |
| Disease of brain, | 0 | 8 | Mania-a-potu, | 2 | 0 |
| " breast, | 0 | 1 | Marasmus, | 3 | 11 |
| " heart, | 8 | 4 | Measles, | 0 | 7 |
| " liver, | 1 | 0 | Mortification of bowels, | 1 | 0 |
| " lungs, | 4 | 1 | Old age, | 15 | 0 |
| " spine, | 0 | 1 | Palsy, | 7 | 0 |
| " throat, | 0 | 1 | Pertussis, | 0 | 9 |
| Dropsy, general, | 7 | 3 | Phthisis pulmonalis, | 49 | 15 |
| " of head, | 0 | 22 | Phlegmasia dolens, | 1 | 0 |
| " heart, | 0 | 1 | Purpura, | 0 | 1 |
| " breast, | 5 | 1 | Rupture of the uterus, | 1 | 0 |
| Drowned, | 0 | 2 | Scalds, | 0 | 1 |
| Dysentery, | 6 | 7 | Scirrhus of stomach, | 2 | 0 |
| Effusion on brain, | 2 | 1 | Scrofula, | 1 | 3 |
| Enlargement of heart, | 1 | 1 | Small pox, | 2 | 3 |
| " liver, | 0 | 1 | Still born, | 0 | 44 |
| Erysipelas, | 1 | 3 | Suicide, | 4 | 0 |
| Fever, catarrhal, | 0 | 2 | Tabes mesenterica, | 0 | 4 |
| " intermittent, | 0 | 1 | Tetanus, | 0 | 1 |
| " puerperal, | 2 | 0 | Tuberculosis, | 1 | 0 |
| " remittent, | 0 | 1 | Tumor, abdominal, | 1 | 0 |
| " scarlet, | 0 | 67 | Ulceration of stomach, | 1 | 0 |
| " typhoid, | 7 | 7 | " throat, | 0 | 2 |
| " typhus, | 6 | 1 | Unknown, | 4 | 10 |
| Gangrene of the lungs, | 0 | 1 | | | |
| Gangrenous inflammation, | 0 | 1 | | 213 | 386 |
| Total, | | | | 599 | |

Of the foregoing the ages were as follows:—

| | | | |
|---|---|---|---|
| Under | 1 year, | | 154 |
| From | 1 | to 2, | 63 |
| | 2 | 5, | 100 |
| | 5 | 10, | 40 |
| | 10 | 15, | 11 |
| | 15 | 20, | 17 |
| | 20 | 30, | 48 |
| | 30 | 40, | 37 |
| | 40 | 50, | 40 |
| | 50 | 60, | 34 |
| | 60 | 70, | 24 |
| | 70 | 80, | 18 |
| | 80 | 90, | 10 |
| | 90 | 100, | 2 |
| | 100 | 110, | 1 |
| | | | 599 |

Included in this number, are 34 from the Almshouse, 47 people of color, and 9 from the surrounding country.

